# Emerging roles of intratumoral microbiota: a key to novel cancer therapies

**DOI:** 10.3389/fonc.2025.1506577

**Published:** 2025-02-25

**Authors:** Pengzhong Fang, Jing Yang, Huiyun Zhang, Diankui Shuai, Min Li, Lin Chen, Liping Liu

**Affiliations:** ^1^ Departments of Emergency Critical Care Medicine, The First Hospital of Lanzhou University, Lanzhou, Gansu, China; ^2^ Department of Gastroenterology, The First Hospital of Lanzhou University, Lanzhou, China; ^3^ The First Clinical Medical College, Lanzhou University, Lanzhou, China; ^4^ Gansu Province Clinical Research Center for Digestive Diseases, The First Hospital of Lanzhou University, Lanzhou, China

**Keywords:** intratumor microbiome, cancer, immune system, gut microbiota, therapy

## Abstract

Microorganisms, including bacteria, viruses, and fungi, have been found to play critical roles in tumor microenvironments. Due to their low biomass and other obstacles, the presence of intratumor microbes has been challenging to definitively establish. However, advances in biotechnology have enabled researchers to reveal the association between intratumor microbiota and cancer. Recent studies have shown that tumor tissues, once thought to be sterile, actually contain various microorganisms. Disrupted mucosal barriers and adjacent normal tissues are important sources of intratumor microbiota. Additionally, microbes can invade tumors by traveling through the bloodstream to the tumor site and infiltrating through damaged blood vessels. These intratumor microbiota may promote the initiation and progression of cancers by inducing genomic instability and mutations, affecting epigenetic modifications, activating oncogenic pathways, and promoting inflammatory responses. This review summarizes the latest advancements in this field, including techniques and methods for identifying and culturing intratumor microbiota, their potential sources, functions, and roles in the efficacy of immunotherapy. It explores the relationship between gut microbiota and intratumor microbiota in cancer patients, and whether altering gut microbiota might influence the characteristics of intratumor microbiota and the host immune microenvironment. Additionally, the review discusses the prospects and limitations of utilizing intratumor microbiota in antitumor immunotherapy.

## 1 Introduction

Cancer is a major societal, public health, and economic challenge in the 21st century (1). According to the World Cancer Report 2022 published by the World Health Organization, there were approximately 20 million new cancer cases and 9.7 million cancer-related deaths in 2022 (1). The human body hosts a diverse community of microorganisms, including viruses, bacteria, fungi, archaea, and unicellular eukaryotes. Remarkably, the number of microbial cells in the human body is significantly greater than the number of human cells (2). These microorganisms have previously been found in various parts of the human body, including the skin, mouth, gastrointestinal tract, respiratory tract, urogenital tract, and other mucosal surfaces (3). With breakthroughs in techniques for detecting low microbial biomass and in-depth research on host-microbe interactions, tissues and organs once thought to be sterile, including the liver, pancreas, lungs, breasts, and kidneys, have also shown the presence of low biomass microbial communities (4). With the deepening research on host-microbe interactions, the concept of intratumor microbiota has been proposed.

**Figure 1:**
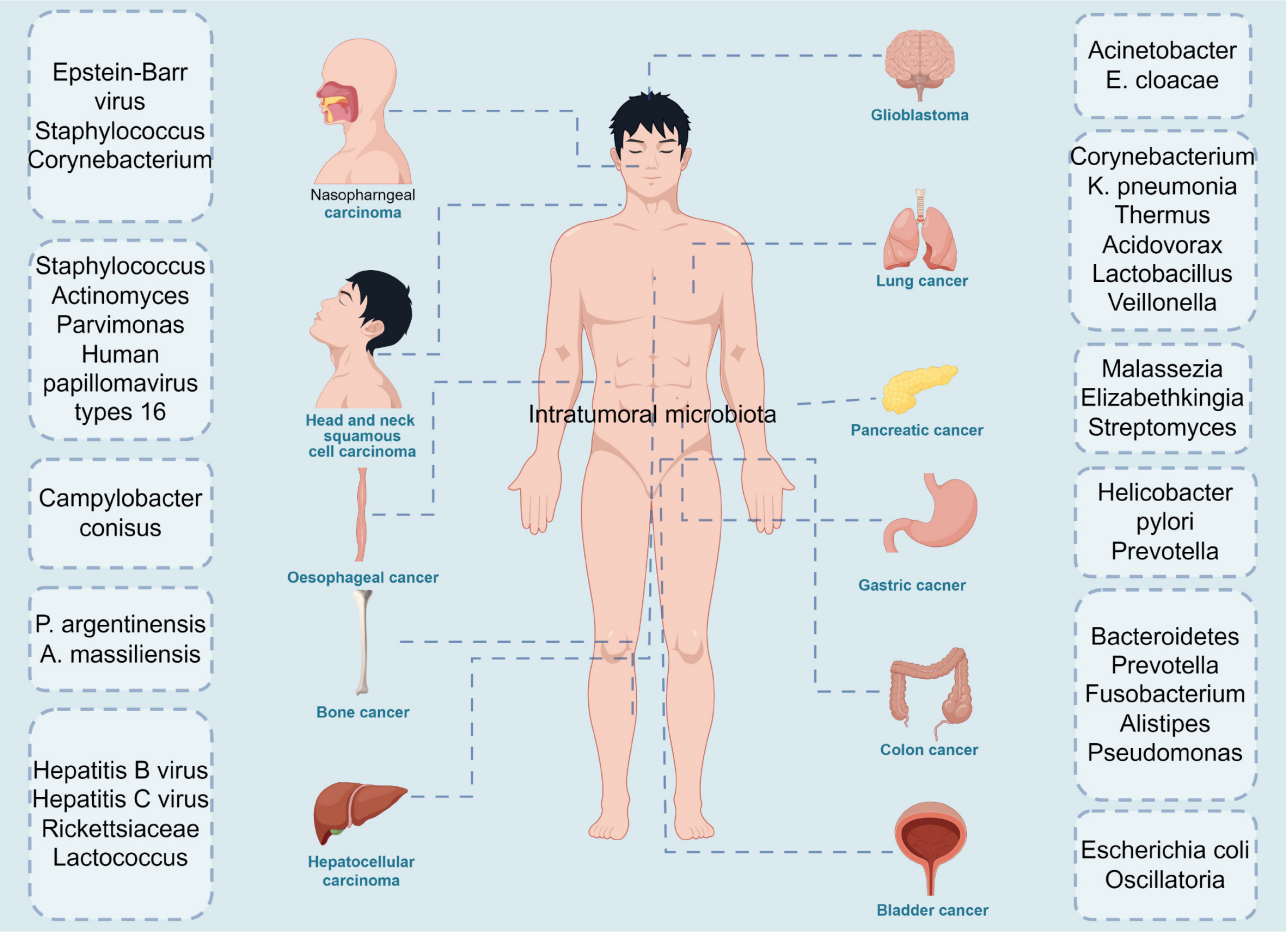
The diversity of intratumoral microbiota in cancers. Microbiota are detected in multiple tumors, including nasopharngeal carcinoma: epstein-Barrvirus, staphylosoccus, corynebacterium, head and neck squamous cell carcinoma: staphylococcus, actinomyces, parvimonas, human papillomavirus type 16, oesophageal cancer: campylobacter conisus, bone cancer: p.argentinensis, a.massiliensis, hepatocellular carcinoma: hepatitis B virus, Hepatitis C virus, Rickettsiaceae, lactococcus, glioblastoma: Acinetobacter, E. cloacae, lung cancer: corynebacterium, K.pneumonia, thermus, acidovorax, lactobacillus, pancreatic cancer: Malassezia, elizabethkingia, streptomyces, gastric cancer: helicobacter pylori, prevotella, colon cancer: Bacteroidetes, prevotella, fusobacterium, alistipes, pseudomonas and bladder cancer: escherichina coli, oscillatoria. The figure was Created by Figdraw.

Notably, recent studies have comprehensively described the cancer mycobiome in 17,401 patient tissue, blood, and plasma samples across 35 cancer types from four independent cohorts, revealing the presence of intratumor mycobiome in these 35 cancer types (5) (**Figure 1**). The gut microbiome is known to play a significant role in tumor development, resistance, and clinical efficacy (6–8). Similarly, the intratumor microbiome has garnered increasing attention for its involvement in tumor diagnosis, prognosis, and its interactions with the immune system (9). Studies indicate that the diversity and composition of the microbiome within tumor tissues influence immune infiltration, ultimately affecting the survival rate of cancer patients (10). Furthermore, the characteristics of the microbiome are associated with cancer type, cancer risk, pathological type, cancer prognosis, and treatment response (10–13). Recent research has revealed that the distribution of the intratumor microbiome is highly organized, featuring immune and epithelial cell functions that promote cancer progression (14). However, the relationship between the gut microbiome and the intratumor microbiome in cancer patients, as well as whether and how bacteria within tumor cells participate in tumor development and influence treatment responses through immune pathways, still requires further in-depth research. In this review, we summarize the relationship between intratumor microbes and cancer development and progression, and highlight the role of intratumor microbiota in tumor immunity and therapeutic responses. We explore the relationship between the gut microbiome and intratumor microbiome in cancer patients, and propose the possibility of applying intratumor microbiota as novel biomarkers and therapeutic targets for cancer.

## 2 Evidence of intratumor microbiota and classic research

In a stable state, commensal microbes have a symbiotic relationship with the host, but microbiome dysbiosis can promote the development of enteritis, pneumonia, and cancer (15). Since the 20th century, people have gradually recognized microorganisms that can induce cancer, such as Epstein-Barr virus, hepatitis B virus, human papilloma virus, and Helicobacter pylori (11). The role of gut microbiota in tumors has been extensively studied, and its influence extends beyond just pathogenesis and cancer risk. Microbial signaling also impacts the clinical course of tumors, including the efficacy, bioavailability, and toxicity of chemotherapeutic and immunotherapy drugs (16). At the same time, advances in detection techniques have provided unprecedented opportunities to study the diversity and functional characteristics of intratumor microbiota (17). Direct detection of intratumor microbes provides the most compelling evidence of their presence within tumor tissues (18). Various advanced detection techniques, including correlative light and electron microscopy (CLEM), fluorescence *in situ* hybridization (FISH) and immunofluorescence (IF), play crucial roles in identifying and studying intratumor microbes. Correlative light and electron microscopy (CLEM) combines the advantages of optical and electron microscopy (19), providing high resolution and environmental context information, making it excellent for detecting intratumor microbiota, but it is costly, time-consuming, and technically complex. Fluorescence *in situ* hybridization (FISH) has strong specificity and can quickly identify and detect various intratumor microorganisms (20), but it has limited resolution and quantification capabilities, and requires specific sample preparation. Immunofluorescence (IF) offers high specificity and sensitivity, allowing real-time observation and multiple staining, making it very effective for detecting intratumor microbiota (21), though it may suffer from fluorescence spectral interference, and is also costly and technically demanding.

In contrast, high-throughput sequencing technologies, such as 16S rRNA gene sequencing and whole metagenome shotgun sequencing (WMS), can provide more comprehensive microbial data. These techniques not only accurately identify and classify microorganisms but also analyze their taxonomic composition and functional characteristics, despite being more complex and expensive (22, 23). By combining these methods, the intratumor microbiome can be studied more precisely and comprehensively. While metagenomics and other methods provide a wealth of data on intratumor microbiota, pure culturing of these microorganisms remains crucial as it reveals their functions and related mechanisms. Significant progress has been made in recent years in culturing previously unculturable microbes. For instance, using methods that simulate natural environments has successfully cultured Candidatus Pelagibacter ubique and other microbes (24). Additionally, a microfluidic intestine-on-a-chip was designed, successfully culturing bacteria from 11 genera, and reverse genomics was used to isolate and culture specific microbes (25). However, the drawbacks of pure culturing include its complexity, high cost, and the difficulty in isolating and successfully culturing low-biomass intratumor microbes. Traditional *in vivo* and *in vitro* experiments, such as flow cytometry, western blot, enzyme-linked immunosorbent assay (ELISA), and mouse models, remain crucial in tumor microbiology research, providing key mechanistic insights by revealing the impact of intratumor microbiota on immune cells and cancer development (18). Omics technologies such as genomics, transcriptomics, and metabolomics are used to study the molecular mechanisms between intratumor microbiota and tumors. Additionally, single-cell RNA sequencing (scRNA-seq) and spatial meta-transcriptomics can elucidate the roles of intratumor microbiota in cancer initiation, progression, metastasis, and treatment responses (26, 27). Other spatial multi-omics techniques, such as spatial genomics, spatial proteomics, and spatial metabolomics, remain indispensable for studying intratumor microbiota and cancer (28). Due to the relatively low biomass of the tumor microbiome, contamination of tumor samples with bacteria or bacterial DNA during sample collection, preparation, and processing can pose significant issues (18). Next-generation sequencing technology acts as a double-edged sword in microbiome research, as its sensitivity can effectively detect both microbial DNA and contaminants, including cross-contamination. Therefore, it is essential to reduce experimental bias and contamination during sampling and processing, include control groups throughout the entire process from sampling to sequencing, and critically assess and report the impact of contamination during analysis (23).

## 3 Origin and diversity of intratumoral microbiota

Despite the considerable attention given to research on intratumor microorganisms, their origins remain to be explored. Tumors have characteristics that make them prone to bacterial colonization: imperfect angiogenesis leads to leaky blood vessels, allowing circulating bacteria to embed; tumors are immune-privileged areas, enabling bacterial proliferation; low-oxygen environments favor anaerobic bacteria; and necrotic regions are nutrient-rich, promoting bacterial growth (29). It is currently believed that intratumor microorganisms mainly originate from the following sources: I) disrupted mucosal barrier sources. Intratumor microorganisms may invade through mucosal barriers, including in gastric cancer, colorectal cancer, pancreatic cancer, lung cancer, and cervical cancer (30). The external cavities of these organs are exposed, and during tumorigenesis, the disruption of the mucosal barrier allows microorganisms colonizing the mucosa to infiltrate the tumor.II) adjacent normal tissues. Research has found a high similarity between the microbiome of tumors and that of their adjacent normal tissues (NAT), leading researchers to propose that intratumor bacteria may originate from NAT (19). However, the source of microbes in NAT is not fully understood and may also originate from the tumor microenvironment. Therefore, more evidence is needed to determine whether NAT is a source of intratumor microbiota. III) Through the blood to the tumor site and infiltrate the tumor through damaged blood. Animal experiments have revealed that certain lymph node metastases (LNMs) resurface at distant metastatic sites through blood vessels in the lymph nodes, rather than through the traditionally assumed lymphatic route (31). In colorectal cancer (CRC), bacteria disrupt the gut vascular barrier (GVB) and disseminate to the liver, inducing the formation of a premetastatic niche and promoting the recruitment of metastatic cells (32). Additionally, studies have shown that different cancer cell clones from the primary tumor can colonize distant organs via the systemic blood circulation, indicating that distant metastases may occur independently of lymph node metastasis (32).

The tumor microbiomes of different cancer types exhibit high heterogeneity (**Table 1**). A large-scale study by Ravid Straussman’s team demonstrated that the microbial composition of each type of tumor (including pancreatic cancer, breast cancer, lung cancer, ovarian cancer, melanoma, brain cancer, and bone cancer) is distinct, with intratumor bacteria primarily found in cancer cells and immune cells (19). Although bacteria belonging to the Firmicutes and Proteobacteria dominate the microbiota of all cancer types, there is a high degree of heterogeneity in the composition and abundance of bacteria in different cancer types, and the composition of microbial species varies in different subtypes of the same tumor type (17). For example, at the species level, Fusobacterium nucleatum was enriched in breast and pancreatic tumors. Saccharomycetes were more abundant in colon cancer, whereas the relative abundance of Malasseziomycetes was higher in melanoma (17). Additionally, the metabolic functions encoded by intratumor bacteria are associated with the clinical characteristics of certain tumor subtypes. For example, enzymes related to anaerobic respiration are more abundant in breast cancer bacteria (19). Bacteria dominate the tumor microbiome, while fungi are less prevalent (5). The study by Galeano Nino et al. further revealed the spatial and population heterogeneity of the intratumor microbiome (14). The microbial distribution also differs between tumor tissues and peritumoral tissues. For instance, the abundance of certain oral microbes is significantly higher in esophageal and gastric cancer tissues than in adjacent normal tissues (65, 66), whereas Fusobacterium nucleatum, which is enriched in colorectal cancer tissues, is not found in adjacent normal tissues (67). An in-depth investigation of the composition and function of the tumor microbiome will provide new opportunities for cancer treatment.

**Table 1:** Characterization of introtumoral microorganisms across different cancers.

Tumor type	Microorganisms	Quantitative dynamics	Ref
Oral cancer	Genus Streptococcus	Decrease	(33)
	Genus Fusobacterium	Increase	(34, 35)
	Pseudomonas aeruginosa	Increase	(36)
	Epstein-Barr virus	Increase	(37)
Oesophageal cancer	Campylobacter conisus	Increase	(38)
	Fusobacterium Nucleatum	Increase	(39)
Gastric cancer	P. stomatis, S. exigua, P. micra, S. anginosus, D. pneumosintes	Increase	(40)
	Proteobacteria, Firmicutes, Bacteroidetes, Actinobacteria, Fusobacteria	Decrease	(41)
	Helicobacter pylori	Increase	(42)
	Streptococcus, Prevotella, Veillonella, Neisseria, Haemophilus	Increase	(43)
Colorectal cancer	Fusobacterium	Increase	(44)
	Bacteroides	Decrease	(44)
	Saccharomycetes	Increase	(5)
	Fusobacterium nucleatum	Increase	(19)
Liver cancer	Hepatitis B virus, hepatitis C virus	Increase	(45)
	Species Helicobacter pylori	Increase	(46)
	Family Streptococcaceae, genus Lactococcus, Gammaproteobacteria	Increase	(47)
	Enterobacteriaceae	Increase	(48)
	Caulobacteraceae, Rickettsiaceae	Decrease	(48)
Pancreatic cancer	Malassezia	Increase	(49)
	Acinetobacter, Pseudomonas, Sphingopyxis	Increase	(50)
	Elizabethkingia	Increase	(7)
Breast cancer	Pseudomonas, Porphyromonas, Azomonas, Proteus	Increase	(51)
	genera Fusobacterium, Atopobium, Gluconacetobacter, Hydrogenophaga and Lactobacillus	Increase	(52)
	enterotoxigenic Bacteroides fragilis	Increase	(53)
	Streptococcaceae, phylum Bacteroidetes	Increase	(54)
	phylum Actinobacteria	Decrease	(54)
Lung cancer	Aggregatibacter, Lactobacillus	Decrease	(55)
	Thermus	Increase	(56)
	Nontypeable Haemophilus influenzae	Increase	(57)
	Veillonella, Megasphaera	Increase	(58)
	Acidovorax, Klebsiella, Anaerococcus	Increase	(59)
Prostatic cancer	P. acnes	Increase	(60)
	Human cytomegalovirus	Decrease	(61)
	Staphylococcus aureus	Increase	(62)
	Cutibacterium acnes	Increase	(63)
	Shewanella	Increase	(64)

## 4 The relationship between intratumor and gut microbiota

Gut microbiota and intratumor microbiota are closely linked in terms of their origins and their impact on immunity. First, intratumor microbiota can originate from gut microbiota because microorganisms from the intestines may be transported through the blood to the tumor site (9). For example, the liver is connected to the gut via the hepatic portal vein. Although the gut vascular barrier restricts the dissemination of intestinal bacteria, when the gut vascular barrier is impaired, intestinal bacteria can promote the recruitment of immune cells in the liver, forming a pre-metastatic niche that supports the metastasis of cancer cells to the liver (17). Studies have found that the gut microbiome can colonize pancreatic tumors in patients with pancreatic cancer, and this colonization can alter the overall microbiome of the tumor (10). Most importantly, both intratumor microbiota and gut microbiota have regulatory effects on the tumor microenvironment. Intratumor microbiota may regulate the host immune response similarly to gut microbiota. Gut microbiota has extensive effects on primary lymphoid organs and adaptive immunity. Studies have shown that gut microbiota significantly impacts immune reconstitution, treatment outcomes, and side effects such as infections following hematopoietic stem cell transplantation (HSCT) (68). Immune reconstitution after allogeneic HSCT is closely related to the diversity of gut microbiota, with higher diversity being significantly associated with lower patient mortality. Moreover, gut microbiota influences the host immune status during homeostasis and tumorigenesis. Cancer therapies have demonstrated strong links between different commensal bacteria and protective antitumor T cell responses. Additionally, probiotics, particularly Lactobacillus and Bifidobacterium, are considered safe (69) and have been used to prevent and treat various diseases (70, 71). In cancer, strains of Lactobacillus and Bifidobacterium can alleviate dysbiosis, enhance antitumor immunity, and improve the efficacy of immune checkpoint inhibitors (72–76). These studies indicate that gut microbiota plays an important role in regulating immune responses and antitumor therapies. Due to the complex interactions between the microbiota and the immune system, numerous studies have focused on how the microbiota influences local and systemic anti-tumor immune responses (72). For example, short-chain fatty acids, such as acetate, butyrate, and propionate, are important energy sources for gut microbiota and play a crucial role in regulating host physiology and immune responses (72).

With further research into intratumor microbiota, it has been found that the tumor microbiome plays a crucial role in reshaping the tumor immune microenvironment (17). When intratumor microbiota is recognized by the innate immune system, the adaptive immune system is activated, becoming a pillar of the antitumor response (77). Tumor microecology helps recruit and activate tumor-supportive immune cells. For example, intratumor microbiota induces IL-17 production, supporting the infiltration of B cells into tumor tissues, thereby promoting colon cancer progression (78). Some intratumor microbiota also counteract pro-tumoral immune responses. For instance, patients with F. nucleatum-positive oral squamous cell carcinoma (OSCC) have lower recurrence rates, less frequent lymph node invasion and metastatic relapse, and longer overall survival (OS), relapse-free survival (RFS), and metastasis-free survival (MFS) compared to those with F.nucleatum-negative tumors (79).Intratumor microbiota can slow cancer progression by enhancing antitumor immunity. For example, gram-negative bacteria were detected in the cytoplasm of osteosarcoma cells and tumor-associated macrophages (TAMs). Patients with localized osteosarcoma had an increased number of antitumor M1 macrophages, which may be related to the abundance of gram-negative bacteria (80). The tumor microbiome plays an essential role in tumor immune regulation, with significant differences in the roles of different microbes in various cancers. Future research should focus on elucidating the interaction mechanisms between intratumor microbiota and the host immune system to develop new cancer treatment strategies.

## 5 Effect of the intratumor microbiota on tumor invasion, metastasis, spatial distribution, and heterogeneity

**Figure 2:**
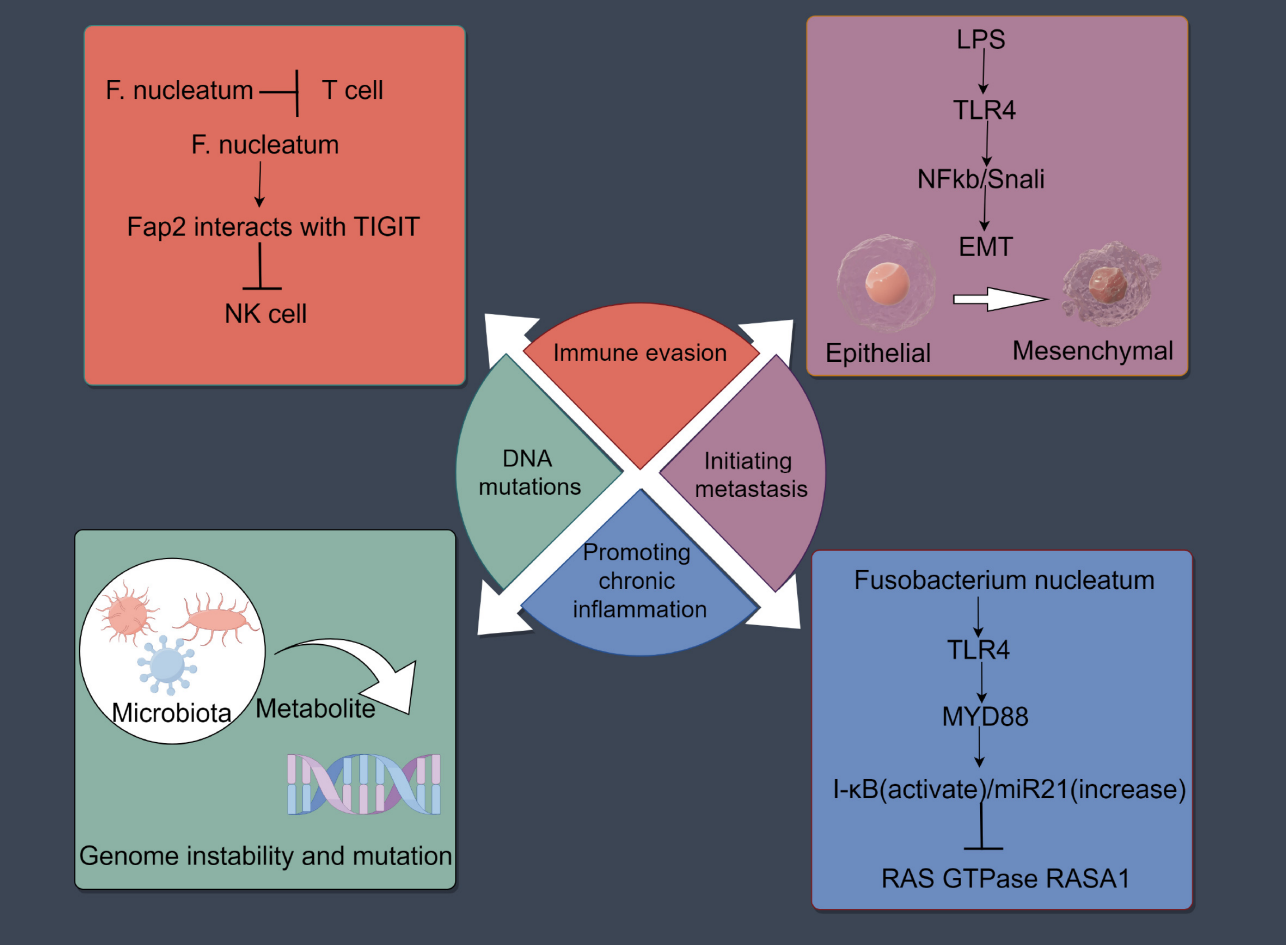
Effects of the intratumoral microbiota on cancers development. Intratumoral microbiota may influence the initiation and progression of cancer through mechanisms such as DNA mutations, immune evasion, promotion of chronic inflammation, and induction of metastasis. The figure was Created by Figdraw.

Increasing evidence has shown that intratumor microbiota can have both positive and negative effects on tumor initiation and progression through mechanisms such as DNA mutations, immune evasion, promoting chronic inflammation, and inducing metastasis (81–84) (**Figure 2**). Importantly, the process of cancer metastasis includes stages such as invasion, dissemination, intravasation, extravasation, and colonization (85). One of the main characteristics of metastasis is its extreme inefficiency; Before successfully reaching and settling in the target organ, cancer cells need to cope with many physical, chemical, and biological challenges (46, 47). Metastasis initiation occurs at an early stage of tumor progression where cancer cells can remotely prepare the pre-metastatic microenvironment (PMN) by secreting components (86). When metastatic cells begin to migrate, they usually invade neighboring tissues en masse to enhance their ability to colonize the new microenvironment (87, 88). Cancer cells usually alter their intrinsic programs to overcome various challenges during metastasis. These programs include the stem cell program/plasticity (for tumor initiation at new sites), the EMT program (for cancer invasion and dissemination), the adhesion program (to prevent apoptosis-induced cell death), and the mechanical stress response program (to resist damage induced by mechanical forces) (89). In addition to directly regulating cancer cells, intra-tumoral bacteria are important inflammatory mediators that shape the specific microenvironment around cancer cells, thus indirectly promoting cancer metastasis (89). Although the number of intratumor microorganisms is low and their biological functions are not yet clear, studies have shown that they are new key players in influencing tumor metastasis and play an important role. For example, studies in breast cancer have found that intratumor bacteria promote host cell survival by reorganizing the actin cytoskeleton, enhancing the resistance of circulating tumor cells to fluid shear stress. The removal of intratumor bacteria can significantly reduce lung metastasis without affecting the growth of the primary tumor (90). Similarly, viable Fusobacterium and its associated microbiota were retained during serial passage in mouse xenografts of human primary colorectal adenocarcinoma (91). Increasing evidence confirms that intratumor bacteria can modulate the intrinsic properties of cancer cells and their external environment, thereby enhancing cancer cell survival and paving the way for cancer metastasis (89) (**Table 2**). Moreover, studies on tumor-associated host-microbiota have primarily relied on bulk tissue analysis, which obscures the spatial distribution and localized effects of microbiota within tumors (14). Research has found that the distribution of intratumor microbiota is highly organized within tumors, clustering in microniches that are less vascularized, highly immunosuppressive, and associated with malignant cells with lower levels of the cell proliferation antigen Ki-67. Bacteria-infected cancer cells invade the surrounding environment as single cells and recruit myeloid cells to bacterial niches. These findings highlight the significant role of intratumor microbiota in promoting cancer progression (14).

**Table 2:** Summary of recent intratumor microbiota studies related to metastasis.

Tumor type	Microbiota type	Effect	Ref
Breast cancer	Staphylococcus, Lactobacillus, and Streptococcus	Promoting tumor metastasis	(90)
Pancreatic cancer	*Fusobacterium nucleatum*	Promoting tumor metastasis	(92)
Colorectal cancer	Escherichia coli	metastasizes to the liver	(32)
Colorectal cancer	H. pylori, Escherichia coli, and *Fusobacterium nucleatum*	translocate into pancreatic tumors	(93)
Bladder cancer	E. coli, butyrate-producing bacterium SM4/1, and Oscillatoria	correlates with EMT	(94)
Pancreatic cancer	Proteobacteria, Bacteroidetes, and Firmicutes	Tumor progression	(7)
Colorectal cancer	Fusobacterium nucleatum	Promoting tumor metastasis	(95)
Oral squamous cell carcinoma	Fusobacterium nucleatum	promotes EMT	(96)

## 6 Intratumor microbiota for treating cancer

Given that local microbial infections can cause tumor regression, the engineering of microbiota to improve and enhance their antitumor effects is being explored (9). The microbiota can serve as bacterial vectors expressing cytotoxic drugs, either alone or in combination with other antitumor agents. Tumor-targeting bacteria demonstrate significant advantages as delivery vectors, including enhanced penetration into tumor tissue, maximized efficacy of chemotherapy agents, and reduced systemic toxicity (8). The microbiota can serve as bacterial vectors expressing cytotoxic drugs, either alone or in combination with other antitumor agents. For example, by coupling the radioisotope 188 Rhenium with attenuated Listeria monocytogenes, a unique radioactive Listeria (RL) was created (97). In normal tissues, Listeria is cleared by the immune system, but in the immunosuppressive tumor microenvironment, it is not. In a highly metastatic pancreatic cancer mouse model, RL effectively delivered radioactivity to metastases without harming normal cells. Multiple low-dose RL treatments significantly reduced the number of metastases (by approximately 90%) (97). Additionally, microbiota can induce both innate and adaptive immune responses against tumor cells. Bacterial therapy can activate the host immune system via multiple mechanisms, including the delivery of cytokines, short hairpin RNA (shRNA), and tumor-associated antigens (98). Engineered bacteria can precisely deliver cytokines and short hairpin RNA (shRNA) to tumor tissues, thereby inducing local inflammatory responses and triggering cytotoxic cell death. Apoptotic or necrotic cancer cells release tumor-associated antigens (TAAs) and various stimulatory factors, promoting the maturation of dendritic cells and triggering epitope spreading effects (98). Additionally, microbiota can induce both innate and adaptive immune responses against tumor cells. The systemic use of potent anticancer drugs such as tumor necrosis factor alpha (TNFα) causes high levels of toxicity and severe side effects. Non-pathogenic Escherichia coli strain MG1655, used as a tumor-targeting system to specifically produce TNFα within tumors in mice, demonstrated the potential of non-pathogenic bacteria as platforms for limiting the activity of potent anticancer agents to tumors (99). At the same time, live microbiota can be used as vectors expressing tumor antigens for tumor vaccination (100). The safety and efficacy of bacterial immunotherapy has always been a priority for clinical applications. Bacterial immunotherapy triggers many of the same potential adverse effects as conventional immunotherapy, including potential systemic immune responses, hypertension and fatigue (98, 101).

Due to the specificity of intratumor microbiota across different tumor types and subtypes, they hold potential as diagnostic tools (**Table 3**). Studies have found that the presence of oral pathogens, Porphyromonas gingivalis and Aggregatibacter actinomycetemcomitans, is associated with an increased risk of pancreatic cancer, while the phylum Fusobacteria and its genus Leptotrichia are associated with a reduced risk of pancreatic cancer (109). Clinical evaluations indicate that tissue and plasma mycobiomes have prognostic and diagnostic capabilities, even in stage I cancers, and exhibit synergistic predictive performance with bacterial communities (5). Some studies have also found that the intratumor microbiota is closely associated with patient prognosis. For example, the prognosis of papillary thyroid carcinoma (PTC) differs between genders and cancer subtypes (13). Further research has found that PTC tissues significantly lack the microorganisms present in adjacent normal tissues, suggesting that these microorganisms are critical in controlling immune cell expression and regulating immune and cancer pathways to slow cancer growth (13). The intratumor load of F. nucleatum may be a potential prognostic factor in stage II/III non-MSI-high/non-sigmoid/non-rectal cancer subset CRC patients receiving oxaliplatin-based adjuvant chemotherapy (110). Therefore, the intratumor microbiota can serve as biomarkers for diagnosis and prognosis.

**Table 3:** The effects of introtumoral microbes on cancer diagnositic/prognostic.

Diagnostic/prognostic	Microbiota	Tumor type	Clinical role	Ref
Diagnositic	Capnocytophaga	Oral Squamous Cell Carcinoma	high accuracy in predicting advanced cancer	(102)
	Fusobacterium	Colorectal cancer	Related to tumor metastasis and progression	(44)
	Acinetobacter, Pseudomonas, Sphingopyxis	Pancreatic cancer	predictive value	(50)
	Porphyromonas gingivalis	pancreatic cancer	contributes to the progression of cancer	(103)
	Veillonella, Megasphaera	Lung cancer	diagnostic biomarker of tumour;	(58)
	Staphylococcaceae	Prostate cancer	Cancer oncogenesis	(104)
Prognosis				
	genus Leptotrichia	Head and Neck Cancer	improve patient prognosis	(105)
	F. nucleatum	colorectal cancer	associated with shorter survival	(106)
	Genus Fusobacterium	Oesophageal cancer	great efficacy in predicting the prognosis	(39)
	Actinomycetales, Pseudomonadales	non-small cell lung cancer	associated with disease-free survival	(107)
	Pseudomonadaceae	Liver cancer	associated with prognosis	(48)
	Helicobacter pylori	Gastric cancer	associated with progression-free survival	(108)

## 7 Conclusions

In conclusion, intratumor microbiota have diverse sources and complex distribution within different tissues, and they are intricately linked with gut microbiota. Intratumor microbiota have potential in the early detection of cancer and in determining patient prognosis. Targeting intratumor microbiota represents a novel clinical approach to cancer treatment, which may improve the efficacy of chemotherapy and enhance the effectiveness of immunotherapy. It is worth noting that although some studies have revealed the mechanisms by which intratumor microbiota influence cancer development, metastasis, and regulation of the tumor microenvironment, the therapeutic effects of microbiota-based treatments may vary depending on tumor type and the immune status of cancer patients. More clinical research is needed to determine their actual value in aiding cancer intervention. Although current experimental technologies have provided us with a deeper understanding of the intratumoral microbiota, we still have limited knowledge of its role in tumorigenesis and progression. This is primarily due to the lack of effective experimental methods to study the occurrence, development, and metastasis of these microorganisms, as well as their spatial distribution and dynamic changes within the tumor.

Although significant progress has been made in recent years in the study of intratumor microbiota, current research still has limitations. For example, the interactions between gut microbiota and intratumor microbiota, and how changes in gut microbiota affect intratumor microbiota and the immune microenvironment, are not well understood. The therapeutic effects and impact of probiotics on cancer and immunotherapy also remain unclear. Further exploration is required to understand the mechanisms through which intratumor microbiota influence antitumor immunity and treatment efficacy, as well as how specific antibiotics can be used to remove immunosuppressive microbes. Future researchers could explore the role of probiotics, antibiotics, and FMT in regulating the intratumoral microbiota in cancer, while dietary patterns also serve as an important modulator of the microbiota.
